# Photoactivated cell-killing amino-based flavylium compounds

**DOI:** 10.1038/s41598-021-01485-y

**Published:** 2021-11-09

**Authors:** Hélder Oliveira, Paula Araújo, Ana Rita Pereira, Nuno Mateus, Victor de Freitas, Joana Oliveira, Iva Fernandes

**Affiliations:** REQUIMTE-Laboratório Associado para a Química Verde, Departamento de Química e Bioquímica, Faculdade de Ciências, Universidade do Porto, Rua do Campo Alegre, 687, 4169-007 Porto, Portugal

**Keywords:** Applied optics, Acne vulgaris, Drug discovery and development

## Abstract

Photodynamic therapy (PDT) is a well-established therapeutic for the treatment of different diseases. The growing interest of this technique required the development of new photosensitizers with better photo-features. This work reports the study of the potential of five nature-inspired amino-based flavylium compounds with different structural features as photosensitizers towards topical PDT. In terms of dark cytotoxicity the five pigments were tested towards confluent skin cells in both fibroblasts and keratinocytes. In the range of concentrations tested (6.3–100 μM), keratinocytes were more prone to growth inhibition and the IC_50_ values for 5OH4′NMe_2_, 7NEt_2_st4′NMe_2_ and 7NEt_2_4′NH_2_ were determined to be 47.3 ± 0.3 μM; 91.0 ± 0.8 μM and 29.8 ± 0.8 μM, respectively. 7NEt_2_4′NMe_2_, 7NEt_2_st4′NMe_2_ and 7NEt_2_4′NH_2_ showed significant fluorescence quantum yields (from 3.40 to 20.20%) and production of singlet oxygen (^1^O_2_). These latter chromophores presented IC_50_ values of growth inhibition of keratinocytes between 0.9 and 1.5 µM, after 10 min of photoactivation with white light. This cellular damage in keratinocyte cells upon white light activation was accompanied with the production of reactive oxygen species (ROS). It was also found that the compounds can induce damage by either type I (ROS production) or type II (singlet oxygen) PDT mechanism, although a higher cell survival was observed in the presence of ^1^O_2_ quenchers. Overall, a structure–activity relationship could be established, ranking the most important functional groups for the photoactivation efficiency as follows: C7-diethylamino > C4′-dimethylamino > C2-styryl.

## Introduction

Topical photodynamic therapy (PDT) is an evolving therapeutic option for the treatment of inflammatory skin diseases such as acne vulgaris, actinic keratosis, psoriasis, sarcoidosis, and several infectious skin diseases and non-melanoma skin cancers^1,2^ and has been demonstrated to be an effective and safe alternative to surgery.

The principle behind PDT relies on the properties of certain dyes (photosensitizers) that have the ability to be photo-activated after their local application or accumulation in the target tissue after systemic administration. Upon the activation of the photosensitizers (PS) with specific light, a series of biochemical processes are initiated leading to the destruction of the target tissue. This may occur by two distinct mechanisms depending on the oxygen pressure at the intracellular level. The irradiation of PS allows its excitation and upon the radiation step, part of the energy is radiated in the form of a quantum of fluorescence while the remaining directs the PS to the triplet state—the bioactive form of the molecule^3^. In this state, the PS can either transfer electrons to the surrounding biomolecules leading to the formation of free radicals and the consequent reaction with oxygen molecules creating reactive oxygen species (ROS) such as superoxide radicals (O_2_^−**·**^) and consequently hydroxyl radicals (HO·) (type I mechanism) or react with ground state oxygen (^3^O_2_) through energy transfer (due to the same spin state), resulting in the production of singlet oxygen (^1^O_2_) (type II mechanism). Both of them result in the production of ROS and given the short lifetime of these species, PDT is able to produce a strong localized effect^4,5^.

Topical PS are mainly used in the field of dermatology as they can be delivered directly to the skin and rarely cause prolonged phototoxicity^6^. Despite the fact that numerous types of PS have been discovered and developed over the years, only a small number of these compounds have been clinically approved being predominantly influenced by the tetrapyrrole structure^7^.

The most commonly used PS in dermatology are precursors of protoporphyrin IX (PpIX), the topical 5-aminolevulinic acid (ALA) and methyl-ALA (MAL). After topical application of the PS precursor, the drug must be metabolized and then the porphyrin accumulates before activation with visible light. PpIX absorbs the greatest amount of light at the 410 nm wavelength, in the blue region. However, PDT light sources more commonly use 630 nm in the red region, giving better tissue penetration. Other PS that have been studied as alternatives to ALA and MAL include hypericin, chlorophyll, indocyanine green (ICG), and indole-3-acetic acid (IAA). Even though porphyrinoid structures comprise the most used PS, several non-porphyrin molecules like anthraquinones, phenothiazines, xanthenes, cyanines, and curcuminoids display photodynamic activity. The main advantage of non-porphyrins PS is that they are used in medicine due to their antibacterial, antiviral, antimicrobial, and staining properties on biological tissues^8^. Moreover, non-porphyrin PS show a maximum absorption wavelength ranging from ~ 420 to 670 nm, but the majority of these chromophores present a maximum absorption wavelength below 600 nm. Thus, there is a current need to apply new classes of compounds to this type of therapy.

A recent study showed that several natural antioxidants have the potential to enhance the efficacy of PDT, probably due to the quenching action driving the mechanism of PDT towards free radical rather than ^1^O_2_^9^.

Anthocyanin’s dual oxidative effect may be explored towards PDT^10^. In fact, due to the reported ability to form quinones and quench singlet oxygen, these pigments may be used in the PDT type I mechanism^11^. Besides anthocyanins, also anthocyanin derived pigments, analogues of pyranoanthocyanins have been proposed as PS^12^.

Based on the knowledge about the current PS, it is possible to optimize the native structures of anthocyanins (absorption in the visible range from 450–530 nm) to yield proper candidates for PDT. For instance, the absorption properties of amino substituents on the 2-phenylbenzopyrylium cores (550–700 nm) may confer them one of the necessary features to be applied in this type of therapy^13^. Also, their photochromic properties were already described^14^.

**Figure 1:**
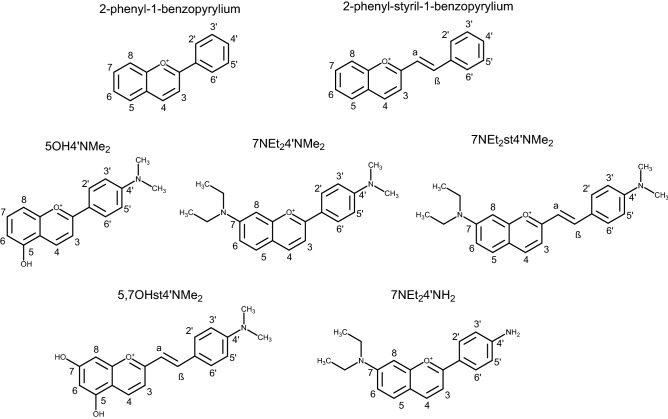
General structure of 2-phenyl-1-benzopyrilium and 2-phenyl-styril-1-benzopyrilium and structure of the amino-based flavylium compounds in their cation form: 5-hydroxy-4′-(dimethylamino)-benzopyrylium (5OH4′NMe_2_); 7-diethylamino-4′-dimethylamino-benzopyrylium (7NEt_2_4′NMe_2_); 7-(diethylamino)-2-(4′-dimethylaminostyryl)-benzopyrylium (7NEt_2_st4′NMe_2_); 5,7-dihydroxy-2-(4′-dimethylaminostyryl)- benzopyrylium (5,7OHst4′NMe_2_); 4′-amino-7-diethylamino-benzopyrylium (7NEt_2_4′NH_2_).

In this study, the suitability of nature-inspired amino-based flavylium dyes (2-phenyl-1-benzopyrylium and 2-phenyl-styril-1-benzopyrilium salts) (Fig. 1) was explored as photosensitizers for PDT by studying the physical–chemical properties of compounds in in vitro model conditions, their cytotoxic effect upon photoactivation in aneuploid immortal keratinocytes from adult human skin and their type of mechanism of action.

## Materials and methods

### Reagents

Singlet Oxygen Sensor Green was purchased from Thermo Fisher Scientific. Dulbecco’s Modified Eagle Medium (DMEM) and foetal bovine serum (FBS) were from Biowest. All the other reagents were from Sigma‐Aldrich, St. Louis, MO, USA.

### Chemical synthesis and purification of the amino-based phenyl and styryl benzopyrylium dyes

5OH4′NMe_2_ was synthesized according to the procedure previously described^15^. Briefly, 2,6-dihydroxybenzaldehyde (1.4 × 10^–3^ mol, 200 mg) and 4′-dimethylaminoacetophenone (1.4 × 10^–3^ mol, 235 mg) were weighted in a round bottom flask and dissolved in 10 mL of a mixture of ethyl acetate: methanol (2:1). Then, chlorotrimethylsilane (TMSCl; 5 equiv) was added and the mixture was left to react at room temperature during 5 h under stirring.

7NEt_2_4′NMe_2_ was synthesized by acidic aldol, condensation between 4-diethylaminosalicylaldehyde and 4′-dimethylaminoacetophenone^13^. 4-diethylaminosalicylaldehyde (1.3 × 10^–3^ mol, 250 mg) and 4′-dimethylaminoacetophenone (2.5 × 10^–3^ mol, 410 mg) were weighted in a round bottom flask and dissolved in 12.5 mL of a mixture of ethyl acetate: methanol (2:1). Then 3.287 mL of chlorotrimethylsilane (TMSCl; 20 equiv) was added and the mixture was left to react at room temperature during 4 h under stirring.

7NEt_2_st4′NMe_2_ was synthesized from the reaction between 4-diethylaminosalicylaldehyde (1.0 × 10^–3^ mol, 200 mg) and 4′-dimethylaminoacetophenone (1.0 × 10^–3^ mol, 200 mg) in 2 mL of acetic acid as reported elsewhere^13^. Chlorotrimethylsilane (TMSCl; 20 equiv) was added and the mixture was left to react overnight at room temperature under stirring.

5,7OH_2_st4′NMe_2_ was obtained from the condensation between 2,4,6-trihydroxybenzaldehyde (1.3 × 10^–4^ mol, 20 mg) and *p*-dimethylaminostyrylmethylketone (1.6 × 10^–4^ mol, 30 mg) in 259 μL (2 mL/1 mmol) of acetic acid and in the presence of hexafluorphosphoric acid (0.5 mL/1 mmol), as previously reported^13^. The mixture was left to react at room temperature during 24 h under stirring.

7NEt_2_4′NH_2_ was synthesized by condensation between 4-diethylaminosalicylaldehyde (5.1 × 10^–4^ mol, 100 mg) and 4′-aminoacetophenone (1.0 × 10^–3^ mol, 140 mg) in 5 mL of a mixture of ethyl acetate: methanol (2:1) in the presence of chlorotrimethylsilane (TMSCl; 20 equiv) at room temperature during 4 h under stirring^13^.

The purity of all compounds was checked by HPLC and NMR.

Stock solution of each flavylium compound (200.0 mM) were prepared in dimethylsulfoxide (DMSO) and kept in the dark at − 18 °C until use.

### HPLC-DAD

Compounds purity and stability were checked by HPLC-DAD (Merck) in a reversed-phase C18 column (Agilent) with 250 × 4.6 mm i.d., particle size 2.7 μm and at 25 °C (Fig. S1). The eluents used were (A) 1% (v/v) formic acid in water and (B) 0.5% (v/v) formic acid in 80% (v/v) acetonitrile and the elution gradient consisted in 40–85% B during 50 min at a flow rate of 0.4 mL/min. After 50 min, the column was washed with 100% B during 10 min and then it was stabilized with the initial conditions for more 10 min.

### Determination of molar absorption coefficient (ɛ)

The molar extinction coefficient or molar absorption (ɛ), of all synthesized pigments was determined in three different solvents (ethanol, methanol and DMSO). For each pigment, in each solvent, a range of concentrations was prepared and then the spectrum of each solution was read. Finally, the respective absorbances (collected at 630 nm) were represented as a function of concentration and the ɛ value was taken from the slope value of the equation curve.

### Fluorescence quantum yields

Based on the absorbance maxima of the different compounds (Fig. S2), the measurement of the quantum yield (QY) of the synthesized flavylium derivatives was performed using a calibrated Quantaurus-QY stand-alone integrating sphere setup C11347-11 (Hamamatsu Photonics, Hamamatsu, Japan) equipped with an integration sphere, a monochromator, a spectrograph, a 150 W xenon light source and a silicon charge coupled device^16^. Each compound was dissolved in DMSO and diluted to ensure an absorbance of 0.1 from the λ_max_ of excitation forward. Each compound was then irradiated at the corresponding wavelength and the absolute quantum yield determined. The fluorescence spectra was also recorded (Fig. S3). For each compound, four independent experiments were performed.

### Oxygen singlet production measurement by Singlet Oxygen Sensor Green

The production of singlet oxygen by the different amino-based compounds in solution was determined as follows: a stock solution of Singlet Oxygen Sensor Green was prepared at 5 mM in methanol (MeOH). Working solutions of the probe were prepared at 2 mM in 100 mM pH 7.5 Tris buffer and in 100 mM pH 7.5 Tris buffer with 10 mM of Sodium Azide. The compounds were then prepared at a stock concentration of 500 μM and the working solutions were prepared in 100 mM pH 7.5 Tris buffer and in 100 mM pH 7.5 Tris buffer with 10 mM of Sodium Azide. 1 μL of Singlet Oxygen Sensor Green working solutions and 100 μL of compounds solution were mixed to a final volume of 1000 μL (in 100 mM pH 7.5 Tris buffer or in 100 mM pH 7.5 Tris buffer with 10 mM of Sodium Azide). 100 μL of each solution was then added to a 96-well black plate and 100 μL of Deuterium Oxide was added for a final concentration of 1 μM Singlet Oxygen Sensor Green and 25 μM for the different amino-based compounds. The fluorescence was then registered in a spectrofluorometer using excitation/emission of 488/525 nm. After this procedure, the wells were irradiated with a white light source with a total of 79.2 J cm^−2^, corresponding to a total irradiation time of 1 h (6 pulses of 10 min) and then, the fluorescence determined using the same excitation/emission pair. As a positive control, Methylene Blue was used at a final concentration of 10 μM. The oxygen singlet production methodology was performed in a dark room.

### Cell culture conditions

Aneuploid immortal keratinocytes from adult human skin, (HaCaT, Cell Lines Service, CLS) were grown as monolayers from passage number 17–43 and maintained at 37 °C in an atmosphere of 5% CO_2_. For routine maintenance, the cells were cultured in 22.1 cm^2^ plates as monolayers and maintained in Dulbecco’s Modified Eagle Medium (DMEM, from CLS), supplemented with 10% foetal bovine serum (FBS, from Biowest) and 1% of antibiotic/antimycotic solution (100 units/mL of penicillin, 10 mg/mL of streptomycin and 0.25 mg/mL of amphotericin B from Sigma‐Aldrich, St. Louis, MO, USA)^17^. The medium was replaced every two days and the cells were harvested when necessary.

### Toxicity to human fibroblasts and keratinocytes

The cytotoxicity of amino-based flavylium compounds to HFF-1 (ATCC Number: SCRC-1041) and HaCaT cells was evaluated using the standard MTT assay^18^. Briefly, cells were seeded at a density of 8 × 10^5^ and 4 × 10^4^ cells/mL, respectively, in 96-well plate and incubated at 37 °C in a 5% CO_2_ atmosphere. Cells were allowed to grow for 24 h or until reaching monolayer confluency, and serially diluted compound solutions (6.3–100 µM) were added to the plate wells. The stock solutions of each compound were prepared at 200 mM in DMSO, the maximum % of DMSO in the cells was 0.05%. Then, cells were incubated for 48 h at 37 °C, after which the plate wells were washed once with phosphate buffered saline (PBS, Sigma-Aldrich), followed by addition of a 0.45 mg/mL MTT solution to each well. Crystals were allowed to form for 1.5 h. The reaction was stopped by rejecting the medium followed by the addition of 100 µL of DMSO (Sigma-Aldrich). The absorbance was read at 570 nm in FlexStation 3 Multi-Mode Microplate Reader (Molecular Devices, California, USA).

### Light source

A white light LED homemade device prototype has 96 LEDs appropriately arranged on a plate of 13 cm length × 8 cm width, with a distance from the microplate surface of 8 cm. The prototype has an irradiance of 21 W m^−2^ and a wavelength range of 408–707 nm (Fig. S4).

The spectral emission of the LEDs system was obtained using a spectrofluorometer (Varian Cary Eclipse, San Diego, CA, USA). The absolute irradiance of the LEDs was evaluated with a Spectroradiometer USB2000 + RAD (OceanOptics, Winter Park, FL, USA)^19^.

## Cellular photoactivation assays

For the cellular photoactivation assays, the white light source of 21 W m^−2^ was used. Briefly, cells were seed at a density of 4 × 10^4^ cells/mL onto 96 –well plates and after 24 h the compounds were added to the plates in different concentrations (6.25–100 μM) and incubated for a period of 4 h at 37 °C in an atmosphere of 5% CO_2_ and in the presence of DMEM with 2% FBS. After this period, the medium with the compounds was removed and fresh medium (DMEM with 2% FBS without phenol red (DMEM_2% FBS_w/o_PR) was added to cells. Following this procedure, cells were submitted to light irradiation, the selected light source at a height of 8 cm, in a chamber designed to ensure the same light quantity for each well during the treatment. Cells were irradiated for 10 min at an intensity of 50%, at room temperature (making a total energy exposition of 6.6 J cm^−2^). The dark treatment was prepared by placing the plates for 10 min in a dark box at room temperature. After this, the 96 well plates were placed in an incubator at 37 °C in an atmosphere of 5% CO_2_ for 24 h. Then, to determine cell survival the Alamar Blue assay was performed as follows: after the 24 h, 20 μL of a solution of Resazurin (0.15 mg mL^−1^ in PBS filtered in 0.22 μm filters) were added to each well (making a total volume of 120 μL per well), and the plates were incubated between 4–6 h (until the development of visible color change from violet to pink). The fluorescence intensity of each well was then determined at 560/590 nm (ex/em) (FlexStation 3 Multi-Mode Microplate Reader).

### Stability and/or metabolization of the amino-based flavylium compounds determined by LC-DAD/ESI–MS

The stability and/or metabolization of the amino-based flavylium compounds in DMEM and DMEM with 2% FBS was studied using LC-DAD/ESI–MS. For each compound, three microtubes were prepared in DMEM and DMEM with 2% FBS and incubated at 37 °C. Each microtube represents a time of incubation (t0 h, t3 h and t6 h) and each sample was analysed immediately after incubation. All compounds were prepared in a concentration of 100 μM. Conditions used in LC–MS analysis were similar to the ones used previously referred for HPLC-DAD, with an injection volume of 20 μL. The spectra were recorder in the positive-ion mode between m/z 150–1000.

### Reactive oxygen species experiments

Cells reactive oxygen species (ROS) production was evaluated following the standard method^18^. Briefly, cells were seed at a density of 4 × 10^4^ cells/mL onto 96 well plates and after 24 h the compounds were added at 25 μM (non-toxic concentration) and the same previous procedure for the photoactivation assays was followed. After that, cells were washed twice in Hank’s Buffered Saline Solution (HBSS) and incubated with 100 μL of 50 μM 2′,7′-dichlorodihydrofluorescein diacetate (DCF‐DA) for 30 min in the same previous conditions. For positive control, cells were treated with 100 μM H_2_O_2_ for 30 min prior to the addition of DCF-DA. Cells were then washed twice with HBSS and incubated with fresh DMEM medium for 24 h. After this period, the fluorescence intensity at 435/585 nm (ex/em) was determined in a FlexStation 3 Multi-Mode Microplate Reader.

### Photodynamic therapy mechanism determination assays

For the determination of the photoactivation mechanism that yielded to cell death, cells were seeded as previously described for the photoactivation assays. After 24 h, compounds at 25 μΜ were added to cells in the presence of different quenchers for type I PDT (l-cysteine and d-Mannitol, both at 10 mM) or type II PDT (Sodium Azide or l-Histidine, both at 10 mM)^20^. The incubation proceeded for a time period of 4 h. After this, solutions containing the compounds were removed from cells and fresh medium containing the different quenchers were added to cells. Cells were irradiated for 10 min at an intensity of 50%, at room temperature (making a total energy exposition of 6.6 J cm^−2^). The dark treatment was done by placing the plates for 10 min in a dark box at room temperature. After this, the medium was removed and replaced by fresh medium without any additive. The 96 well plates were then placed in an incubator at 37 °C in an atmosphere of 5% CO_2_ for 24 h. Finally, the Alamar Blue assay was performed to determine cell survival as previously described.

### Uptake determination assays

#### Overall uptake

The cellular uptake of the different compounds by HaCaT cells was determined following the protocol: cells were seeded at a density of 4 × 10^4^ cells/well onto 12-well plates (1060 μL per well) and after 24 h the compounds were added to the plates in different concentrations (6.25–100 μM; 1060 μL per well) and incubated for a period of 4 h at 37 °C in an atmosphere of 5% CO_2_ and in the presence of DMEM with 2% FBS. Then, the medium with the compounds was removed, cells are washed with PBS to remove any extracellular compounds and the same volume of DMSO (1060 µL) was added to cells. After 60 min in the dark, the plates were submitted to fluorescence determination. The fluorescence intensity of each well was then recorded at the different wavelength pairs specific for each tested compound (ex/em) in a FlexStation 3 Multi-Mode Microplate Reader. The fluorescence values were then used to plot into the corresponding calibration curves and the uptake amounts were determined.

- Fl_577/658_ = 19.734 × Conc (7NEt_2_4′NMe_2_), μM; Fl_577/769_ = 0.2869 × Conc (7NEt_2_st4′NMe_2_), μM;
- Fl_577/635_ = 24.556 × Conc (7NEt_2_4′NH_2_), μM; Fl_577/635_ = 42.851 × Conc (PpIX), μM;

#### Adsorption/absorption ratio

The ratio between the absorption and surface adsorption by HaCaT for each compound was performed. Cells were seeded in the same conditions as previously stated. After 24 h, compounds were added to the plates at the concentration of 100 μM (1060 μL per well) and incubated for a period of 4 h at 37 °C in an atmosphere of 5% CO_2_ and in the presence of DMEM with 2% FBS. After incubation, the medium with the compounds was removed and Dulbecco’s phosphate buffer (DPBS) was added (1060 μL per well). The fluorescence intensity of each well was then determined at the different wavelength pairs specific for each tested compound (ex/em) using a FlexStation 3 Multi‐Mode Microplate Reader. After this, 300 μL of Trypan Blue was added (making a total of 1060 μL) to each well and the fluorescence intensity was determined in the same conditions^21^. Cells with DPBS and cells with DPBS + Trypan Blue were used as controls.

- Fl_577/658_ = 19.734 × Conc (7NEt_2_4′NMe_2_), μM; Fl_577/769_ = 0.2869 × Conc (7NEt_2_st4′NMe_2_), μM;
- Fl_577/635_ = 24.556 × Conc (7NEt_2_4′NH_2_), μM; Fl_577/635_ = 42.851 × Conc (PpIX), μM;

#### Overall kinetics uptake

The cellular uptake kinetics of the different compounds by HaCaT cells was determined as follows: cells were seeded at a density of 4 × 10^4^ cells/well onto 12-well plates (1060 μL per well) and after 24 h compounds were added to the plates at a concentration of 100 μM (1060 μL per well) and incubated for different time periods (15, 30, 60, 120, 180 and 240 min) at 37 °C in an atmosphere of 5% CO_2_ and in the presence of DMEM with 2% FBS. After each time period, the medium with the compounds was removed and DMSO was added to cells. After 60 min in the dark the plates were submitted to fluorescence determination. The fluorescence intensity of each well was then determined at the different wavelength pairs specific for each tested compound (ex/em) (FlexStation 3 Multi-Mode Microplate Reader). The fluorescence values were then used to plot into the corresponding calibration curves and the uptake amounts were determined.

## Results and discussion

Flavylium cations are 2-phenyl-1-benzopyrylium derivatives that constitute the structural core of anthocyanins. In moderately acidic aqueous solutions, flavylium compounds undergo a pH-dependent network of reversible chemical reactions. This network can be described as a single acid–base reaction (only for the compounds having hydroxyl substituents) involving a flavylium cation (acidic form) and a mixture of basic forms (quinoidal base (only for the compounds having hydroxyl substituents), hemiketal and *cis* and *trans* chalcones)^22^. While most flavylium cation no longer exist at these pH values, the introduction of amino groups in adequate positions of the 2-phenyl-1-benzopyrylium core delocalizes the positive charge of the pyrylium ring and extends the pH range of the flavylium stability to the neutral region (Fig. S5)^23^. Three compounds with 2-phenyl-1-benzopyrilium core were synthesized including 5-hydroxy-4′-(dimethylamino)-benzopyrylium (5OH4′NMe_2_); 7-diethylamino-4′-dimethylamino-benzopyrylium (7NEt_2_4′NMe_2_) and 7-diethylamino-4′-amino-benzopyrylium (7NEt_2_4′NH_2_).

Besides this chemical stability improvement associated with the inclusion of amino groups, another explored possible strategy to achieve colour and stability at the same time is the extension of the π conjugation of the flavylium by introducing a double bond between ring C and ring B as in 2-styryl-1-benzopyrylium (commonly referred as styrylflavylium). Two compounds with 2-phenyl-styril-1-benzopyrilium core were obtained by chemical synthesis 7-diethylamino-2-(4′-dimethylaminostyryl)-benzopyrylium (7NEt_2_st4′NMe_2_) and 5,7-dihydroxy-2-(4′-dimethylaminostyryl)-benzopyrylium (5,7OHst4′NMe_2_).

Since the five compounds are structurally related, it will be possible to establish a structure–activity relationship aiming to determine the most relevant structural features to meet the requirements to be an ideal PS.

These chromophores have to show strong absorption in the red region (600–800 nm, therapeutic window), since this high absorption of red light by these amino-based flavylium compounds in the therapeutic window will result in maximum penetration of light into the tissues. In fact, and compared to that of proto-porphyrin IX (a well-known photosensitizer), the molar absorption coefficient (ɛ) in DMSO determined at λ 630 nm (maximum wavelength for red light) of the five compounds is greatly improved, with 7NEt_2_st4′NMe_2_ and 5,7OHst4′NMe_2_ being the ones with the higher values (Table 1). Among, the five dyes the molar absorption coefficient (ɛ) in alcoholic solutions was higher for 5,7OHst4′NMe_2_ and 7NEt_2_st4′NMe_2_, the only pigments having at the same time an amino and a styryl group. Ideally this feature has to be coupled with other, high singlet oxygen production, to conceptualize an ideal PS, as will be evaluated in next section.

**Table 1:** Molar absorption coefficient (ɛ) for the different compounds by measuring absorbance at λ = 630 nm (maximum wavelength for red light).

Compound	ɛ (M^−1^ cm^−1^) at 630 nm		
	DMSO	Methanol	Ethanol
5OH4′NMe_2_	ND	ND	ND
7NEt_2_4′NMe_2_	11,031	8,720	10,725
7NEt_2_st4′NMe_2_	15,728	22,749	16,281
5,7OHst4′NMe_2_	15,224	15,224	16,422
7NEt_2_4′NH_2_	5687	1682	2225
PpIX^1^	3207	–	–

^1^For PpIX the determination was only performed in DMSO, due to solubility restrictions.

### Fluorescence quantum yields (Φ_f_) and oxygen singlet production

Knowing that the triplet state of dyes is an important feature for PDT mechanism and that there is an inner relation with fluorescence parameters, it is important to evaluate whether a certain candidate dye has potential to emit fluorescence, revealed by their fluorescence quantum yield (Φ_f_). When irradiated by specific wavelengths, a molecule is converted to its excited single states (S_1_ or S_2_). Upon reaching these higher energy levels, the molecule can return to its normal state (S_0_) directly from S_1_ or from S_2_ via internal conversion to S_1_ by emitting fluorescence. However, in the S_1_ state the molecule can also be submitted to an intersystem crossing where it is converted to its triplet state (T_1_).

The determination of the fluorescence quantum yields for the different amino-based flavylium compounds by the absolute method revealed that Φ_f_ are structure related. Table 2 shows that these values varied from 0 to 20% with 5OH4′NMe_2_ and 5,7-OHst4′NMe_2_ showing undetectable values, while 7NEt_2_4′NH_2_ presented the highest value of Φ_f_ (20.20 ± 0.47%). 7NEt_2_4′NMe_2_ and 7NEt_2_st4′NMe_2_ presented 5.83 ± 0.28% and 3.40 ± 0.17%, respectively.

**Table 2:** Quantum yields of fluorescence (Φ_f_) for the different compounds determined by absolute method using an integrating sphere.

Compound	$\overline{f} \pm SD$(%)
5OH4′NMe_2_	N.D.
7NEt_2_4′NMe_2_	5.83 ± 0.28
7NEt_2_st4′NMe_2_	3.40 ± 0.17
5,7OHst4′NMe_2_	N.D.
7NEt_2_4′NH_2_	20.20 ± 0.47

**Figure 2:**
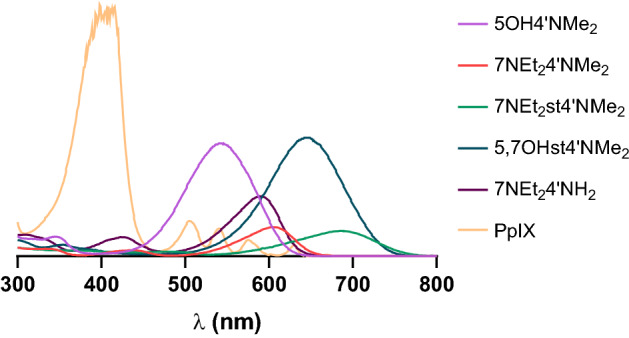
UV–Vis absorption spectra of the different synthetized amino-based flavylium compounds determined at pH 5.5 in 1% DMSO.

Moreover, it was possible to correlate the structural features of compounds (Fig. 1) with Φ_f_. Concerning the fluorescence quantum yield of compounds, in all compounds without the diethylamino group at C7 and with OH groups at ring A, the fluorescence could not be detected. In order to understand, if the structural requirement was the presence of amino groups or the lack of OH groups, one pigment with the diethylamino group at C7, but having an OH at C4′ was tested (data not shown). In this case no fluorescence signal was observed after exciting at its maximum absorption wavelength. This proves the association of the lack of OH groups to the ability to emit fluorescence. On the other hand, the primary amine group at C4′ seems to highly contribute to the excitability of the molecule, while the styryl (by comparing 7NEt_2_4′NMe_2_ and 7NEt_2_st4′NMe_2_) seems to reduce this property. From comparison with PpIX, a widely known PDT photosensitizer with a fluorescence quantum yield of 8.5%^24^, the present compounds also display significant values, including 7NEt_2_4′NH_2_ that displayed a much higher yield. Furthermore, the Φ_f_ seems to be related with the absorption maxima at higher wavelengths (Fig. 2).

However, it is important to have in mind that the fact that a compound emits fluorescence does not imperatively mean that it will have a partial triplet state. Consequently, to assess the role in PDT, other methods must be implied, namely the ability to produce oxygen singlet and their direct action on cellular models upon light irradiation.

**Figure 3:**
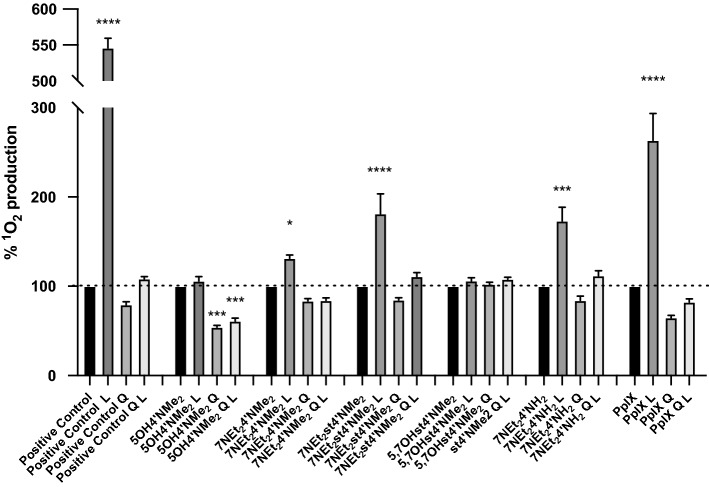
Oxygen singlet production by the compounds through Singlet Oxygen Sensor Green assay. The compounds were prepared in the different solutions at a final concentration of 25 μM. The fluorescence was registered before and after light irradiation (79.2 J cm^−2^) at ex/em 488/525 nm. As positive control methylene blue at 10 μM was used. *L* result after light irradiation; *Q* result in the presence of the quencher NaN_3_ 10 mM; *QL* result after light irradiation in the presence of the quencher NaN_3_ 10 mM. Each value represents the mean ± SEM (n = 8–16). *Means significantly different to the treatment control with the white light (****p value < 0.0001, ***p value < 0.001, *p value < 0.05). To perform both statistical analysis, ordinary one-way ANOVA with multiple comparisons between all means of each column was performed.

With that in mind, the compounds were subjected to photoactivation in the presence of a specific probe that is highly selective for ^1^O_2_. From Fig. 3 is possible to observe the compounds showed different activities on oxygen singlet production upon light irradiation. 5OH4′NMe_2_ and 5,7OHst4′NMe_2_ showed non-significant enhancement on ^1^O_2_ production, contrarily to the other three amino-based flavylium compounds. For 7NEt_2_4′NMe_2_ was observed a 131.2 ± 3.90% ^1^O_2_ production and for 7NEt_2_4′NH_2_ and 7NEt_2_st4′NMe_2_, the values were even higher, 173.1 ± 15.73 and 181.2 ± 22.23%, respectively. The first important point to highlight is the lack of singlet oxygen generation by the two compounds having hydroxyl groups in their structure. The three ones that were able to produce singlet oxygen, have in their structure a dimethyl amino group at C7 and an amine at C4′. Within these three compounds, and comparing 7NEt_2_4′NMe_2_ and 7NEt_2_4′NH_2_ structure, the primary amine seems to favor the production of singlet oxygen. It is also possible to conclude that the inclusion of the styryl group is also favorable (comparing compound 7NEt_2_4′NMe_2_ with compound 7NEt_2_st4′NMe_2_). The above highlights are supported by the similar effects of both primary amine and styryl groups (7NEt_2_st4′NMe_2_ versus 7NEt_2_4′NH_2_).

The compounds were stable to light irradiation and a cumulative ^1^O_2_ production was observed. This fact is indicative of the absence of degradation of the PS by the generated ^1^O_2_. As a positive control, methylene blue was used at a concentration of 10 μM and for the same amount of light irradiation the ^1^O_2_ production yield was about 5-folder higher, which ensured the success of the experiment. On the other hand, PpIX was also tested in the same conditions as the compounds. The results showed an improvement of 263.3 ± 12.28% on the production of ^1^O_2_.

Although the compounds were clearly worst comparing to PpIX, significant differences were found on the production of singlet oxygen before and after light irradiation. Furthermore, a similar pattern can be found when comparing the cellular experiment in the presence of type II quenchers. This reinforces the role of these compounds as potential photosensitizers and their involvement in type II PDT mechanism. Sodium azide was used to corroborate the ^1^O_2_ production as it is a well-known quencher for this molecule. It is possible to observe a clear reduction on the ^1^O_2_ amount upon light irradiation in the presence of NaN_3_, suggesting an efficient quenching. Interestingly, for 5OH4′NMe_2_ the reduction was significant even when compared to the dark control, suggesting that this compound can act as pro-oxidant without light activation.

### Cytotoxicity assays in proliferation and in confluent cells

Chronic lesions of psoriasis and atopic dermatitis are remarkably similar with respect to keratinocytes cellular proliferation^25^. Besides other pathognomonic changes, both skin lesions are characterized by an abnormal proliferation and differentiation of keratinocytes leading to epidermal hyperplasia^26^.

To understand the selectivity of the amino-based phenyl and styryl benzopyrylium compounds to normal and hyperproliferating cells, the cytotoxicity of compounds was evaluated in a range of concentrations from 6.3–100 µM during 48 h. This period of time was established not only to follow standard procedures but was also based on the possible permeation and accumulation of compounds in skin tissue, besides the short period of incubation normally performed in clinical procedures.

**Figure 4:**
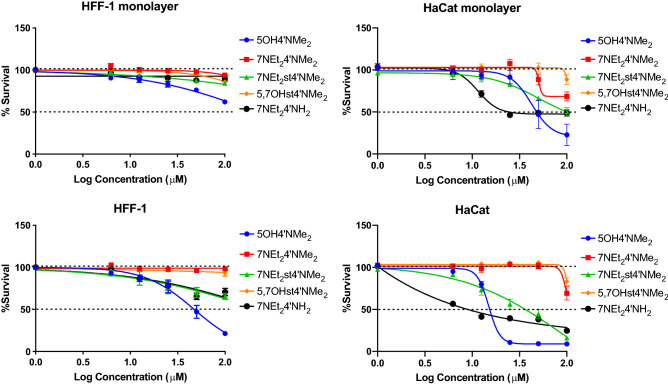
Effect of compounds on HFF-1 and HaCaT cells proliferation evaluated by the MTT assay. Both cell lines were seeded in 96 well plates and then treated with a concentration range (0.00–100.00 µM) of each compound for 48 h. Each value represents the mean ± SEM (n = 4–12).

Overall, the results showed a similar pattern for both cell lines, although in the case of monolayer confluency, the compounds presented less cytotoxicity for both cell lines when comparing to cells in proliferation, especially for the higher concentrations tested (Fig. 4).

In proliferation, cells are highly active as they have several overexpressed growing genes and may be more prone to suffer the effects of external factors, while in monolayer, such propensity may be diminished. 5OH4′NMe_2_ showed to be the most cytotoxic compound for both cells. It showed a significant reduction on cells survival for the two conditions tested in both cell lines. This was followed by 7NEt_2_st4′NMe_2_ and 7NEt_2_4′NH_2_. Another evident observation is the higher resistance of HFF-1 cells when compared to HaCaT. In the case of HFF-1, only 5OH4′NMe_2_ presented an IC_50_ value (47.25 ± 0.2103 μM) in the range of the concentrations tested, for the proliferation condition, while in HaCaT, 5OH4′NMe_2_, 7NEt_2_st4′NMe_2_ and 7NEt_2_4′NH_2_ presented IC_50_ values in the two tested conditions (14.9 ± 0.1 μM; 35.42 ± 0.02 μM; 8.9 ± 0.2 μM for proliferation and 47.3 ± 0.3 μM; 91.0 ± 0.8 μM; 29.8 ± 0.8 μM for monolayer confluence). Such observation suggests a specificity for the different cell phenotypes which in the context of PDT is crucial for the success of the bioaccumulation in the damage tissues.

We propose a tentative structure–activity relationship for the compounds tested for both cell lines (although less pronounced in the case of HFF-1). The presence of the primary amine group at C4′ also seems to have an important role for the toxicity of the phenyl benzopyrilium structure. However, in the case of the styryl benzopyrylium, the results are not clear about the contribution of the double bound for the cytotoxicity on the experimental conditions tested. Such differences may be due to the inner rearrangements of the structures in solution, and the hindrance effects of the key functional groups. Although much less evident, the diethylamino and dimethylamino groups in C7 and C4′ positions may also contribute to the toxicity role of the benzopyrylium core structure. Based on these results, the next experiments were only performed in the HaCaT cellular model.

### Photoactivation experiments

For the photoactivation experiments, HaCaT cells were selected as an appropriate skin barrier model to evaluate the potential of the different compounds for PDT. For this, compounds were incubated during 4 h at different concentrations (applied concentration) with HaCaT proliferating cells, then they were removed and replaced with fresh medium, and after that, plates were either irradiated with white light with a total energy of 6.6 J cm^−2^ or maintained in the dark at room temperature during the corresponding time of irradiation. The selection of the light source was based on the fact that the compounds have specific absorption spectra with different maxima (Fig. 2), thus using a white light ensures irradiation at all the maxima for a proper photo-activation of compounds. From Fig. S6, it is possible to observe differences between the responses of the different compounds in presence and absence of light. For these experiments, protoporphyrin IX, a widely used molecule for skin PDT, was used to optimize the experimental conditions. 5OH4′NMe_2_ showed a significant toxicity for keratinocytes cells either in the presence or absence of light reaching a value of 40% of survival at the highest concentration tested. We also observed significant differences between dark and light treatments for the lowest concentrations tested. However, in the cases that no significant cytotoxicity was induced by 5OH4′NMe_2_ (at applied concentration range 6.25 and 12.5 μΜ) in the dark, upon light irradiation the cells survival reduced less than 20%. Also, for applied concentrations higher than 25 μΜ, both dark and light presented cytotoxicity and for the two highest concentrations applied the light treatment did not present any effect on the reduction of the cell survival. Such results suggest that this compound may have a small to moderate action at lower concentrations where it is not cytotoxic unless light stimulus occur. This result is in agreement with the previous results for the 48 h cytotoxicity, confirming a high toxic effect of this compound on cells. Regarding 7NEt_2_4′NMe_2_ it was possible to observe that within the range of concentrations initially applied, no significant decrease on cell survival was observable in the absence of light, which indicates the absence of dark cytotoxicity. However, upon white light irradiation, the survival of cells highly decreased in a concentration dependent manner, reaching a minimum of 38% for the highest concentration of 7NEt_2_4′NMe_2_. Significant differences were found for applied concentrations above 6.25 μΜ between dark and white light irradiation, indicating a photoactivation even for low concentrations. With respect to 7NEt_2_st4′NMe_2_, similar results were observed, however, for the higher concentrations applied (50 and 100 μM) this compound present a significant dark cytotoxicity in comparison to control, and consequently a significant photoactivation was found for the last four concentrations. 5,7OHt4′NMe_2_ did not present significant effects in the survival rate of HaCaT cells both in the absence and presence of light, which suggests that this compound is not photoactivated or toxic for HaCaT cells. Concerning 7NEt_2_4′NH_2_, only for the higher concentrations tested it is possible to see significant differences between dark and light irradiation, thereby indicating a potential photo-activation in these conditions. Nevertheless, for the higher concentration applied a significant decrease of cell survival is observed in dark conditions. PpIX was used as a control experiment, as the results show an effective photoactivation from the three higher concentrations beyond with no cytotoxic effects in dark conditions.

However, it is important to have in mind that the design of the experiment consists in the removal of the compounds after 4 h of incubation and prior to light/dark treatments. This intends to mimic the effective absorption of the compounds towards the skin barrier. Consequently, the concentration of compound that will be effectively photoactivated will be different from those initially applied. To assess this, the study of the uptake of the most promising compounds (7NEt_2_4′NMe_2_, 7NEt_2_st4′NMe_2_ and 7NEt_2_4′NH_2_) by the cellular model was performed and the results showed that the uptake of the compounds by HaCaT cells model is dependent on the structure (Table 3).

**Table 3:** Percentage of compounds absorbed/adsorbed by HaCaT cells after 4 h of incubation for the different concentrations of compounds. The results are presented in % ± SEM (n = 4–6).

Initial Concentration (μM)	% Uptake ± SEM			
	7NEt_2_4′NMe_2_	7NEt_2_st4′NMe_2_	7NEt_2_4′NH_2_	PpIX
6.25	3.416 ± 0.629	4.448 ± 0.876	1.229 ± 0.296	0.694 ± 0.110
12.5	4.156 ± 0.764	4.236 ± 0.628	1.828 ± 0.419	0.615 ± 0.050
25	2.745 ± 0.504	3.564 ± 0.613	1.977 ± 0.581	0.508 ± 0.021
50	3.660 ± 0.831	4.091 ± 0.552	2.192 ± 0.662	0.667 ± 0.040
100	3.523 ± 0.695	5.830 ± 1.569	1.785 ± 0.383	0.659 ± 0.037

Also, in the experimental conditions tested, the concentration does not seem to affect the rate of the uptake during the 4 h period as no significant differences were found among the several initial concentrations applied for the different compounds.

**Figure 5:**
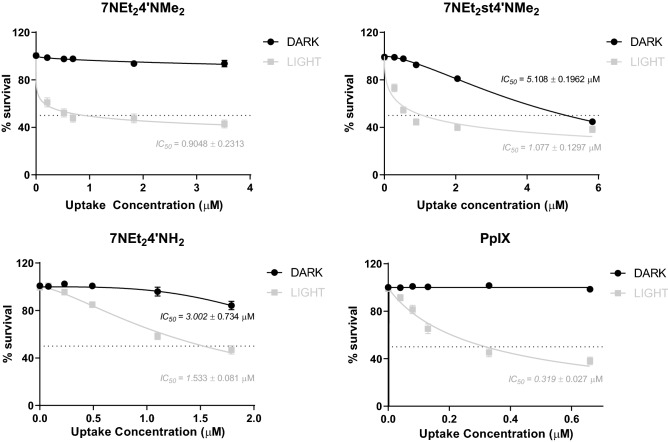
HaCaT cells survival in the presence of the most promising compounds upon irradiation with white light (6.6 J cm^−2^) or maintained in the dark. As a control for the experiment, protoporphyrin IX (PpIX) was used. The results are presented as the logarithmic function of the different tested concentrations. Each value represents the mean ± SEM (n = 16–32).

For 7NEt_2_4′NMe_2_ the uptake amount after the 4 h incubation period ranged between 2.745–4.156% and for 7NEt_2_st4′NMe_2_ the range was between 3.564–5.830%. On the other hand, 7NEt_2_4′NH_2_ presented a much lower uptake ranging between 1.229–2.192%. PpIX was the lowest to be uptake in comparison to the initial concentrations applied, ranging between 0.459–0.694%. These results suggest that indeed the concentrations of compound necessary for an effective action after photoactivation are much lower than the initial applied concentrations, which for an applicability perspective is of high importance. In summary, the IC_50_ for 7NEt_2_4′NMe_2_, 7NEt_2_st4′NMe_2_ and 7NEt_2_4′NH_2_ after light exposure ranged between [0.9–1.5 µM], with PpIX presenting an in the same range (IC_50_ = 0.3 µM) (Fig. 5).

It is important to notice that these results do not distinguish between the amount absorbed into the cells and the amount that may be adsorbed at the surface of the cells. To understand the ratio between internalized/surface adsorbed compounds trypan blue was used. In healthy cells, trypan blue is not able to penetrate cell membrane, thereby it may act as a quencher with the adsorbed compounds at the cells surface withdrawing their fluorescence intensity. In this case, by measuring the fluorescence intensity at the specific pairs of each compound before and after the application of trypan blue to the wells, it is possible to determine this ratio. From Fig. S7, it is possible to observe that the majority of compounds are adsorbed at the surface of the cells instead of being effectively internalized. However, no correlation was found between the amounts of internalized/adsorbed for the different compounds and their photoactivation. This suggests that compounds adsorbed at the cell surface may also be involved in PDT mechanisms. Furthermore, a kinetic of the uptake was also performed to understand if this parameter was dependent on the structure of the compounds. The results showed that for all compounds, after 120 min of incubation, the concentration of compound that is internalized by cells seem to stabilize (Fig. S8). This suggest that the kinetics is dependent on the number of cells rather than on the structure of the compound. However, it is important to have in mind that the total uptake is different in each compound (Table 3). These results are important from a therapeutical perspective, meaning a reduction of the waiting time for the patient with a similar efficacy. However, the 4 h time period was initially applied to ensure a proper mechanistic study for each compound tested.

By integrating these results, the photoactivation seems to depend on specific structural features which may be ranked as follows: C7-diethylamino > C4′-dimethylamino > C2-stryryl. Furthermore, the styryl function seems to induce an inner cytotoxicity to the structural features of the compounds in dark conditions. The same can be observed to the C4′-NH_2_ function, while in a more discrete extent. These results can be partially correlated with the 48 h toxicity assay, particularly for the dark condition. Altogether, they suggest that the incubation period is a key factor for the effect of the compounds and for PDT application, since too much time results in undesired toxic effects without photoactivation.

### Stability/metabolization of the amino-based flavylium compounds

A PS agent suitable for PDT must present stability in the solvents in which it is irradiated, having at the same time low dark degradation and also low photobleaching to prevent its degradation during the therapy.

In order to evaluate this feature, the stability/metabolization of the most promising amino-based flavylium compounds (7NEt_2_4′NMe_2_, 7NEt_2_st4′NMe_2_, 7NEt_2_4′NH_2_) was studied in DMEM with or without 2% FBS during 6 h. For all compounds incubated in DMEM, the results obtained showed a decrease in the percentage of each compound over time (Fig. S9). Although part of this decrease is attributed to the conversion of the flavylium cation into the chalcone form and dimerization (Fig. S9), a fraction is attributed to chemical degradation by temperature and pH (Fig. S9).

**Figure 6:**
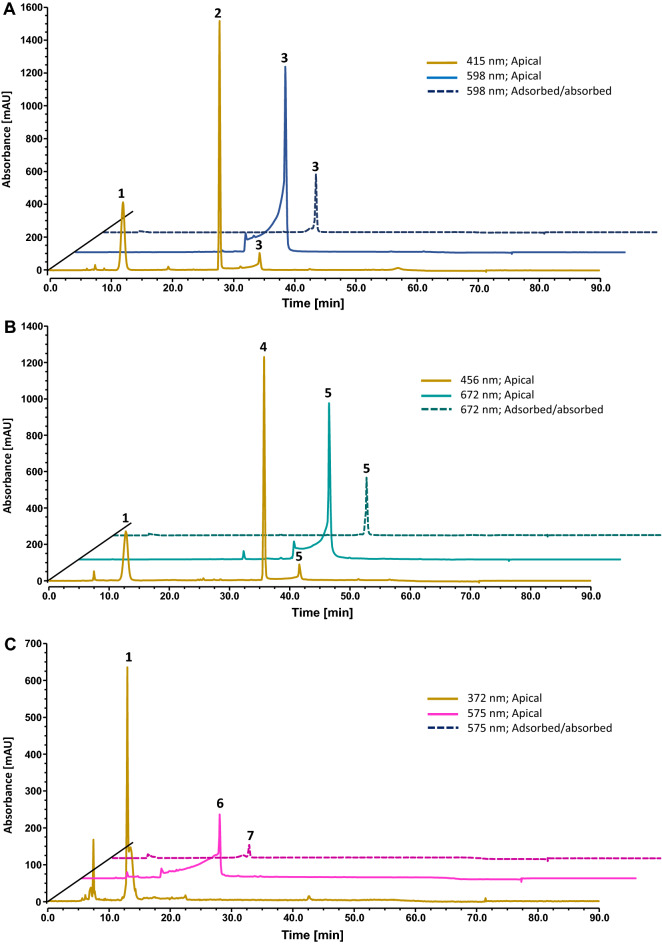
Chromatographic representation of (**A**) 7NEt_2_4′NMe_2_ (100 μM), (**B**) 7NEt_2_st4′NMe_2_ and (**C**) 7NEt_2_4′NH_2_ in the studies of the cellular uptake: compounds species present at the apical side in DMEM 2% FBS after 4 h in contact with HaCaT recorded at chalcone form maximum wavelength and at flavylium form maximum wavelength. Compounds forms adsorbed/absorbed by HaCaT cells after 4 h of incubation, at their maximum wavelength are presented as a dotted line. At the bottom of the figure is summarized the tentatively identification of all peaks by LC-DAD/ESI–MS analysis.

Regarding the incubation of the compounds in DMEM with 2% FBS, it was also observed a decrease in the amount of each compound over time (Fig. S9). The presence of 2% FBS led to a higher conversion between the flavylium cation form and the respective chalcone form for all the compounds studied (Fig. S9). The higher degradation of each compound over time is attributed to the enzymatic degradation resulting from the presence of FBS (Fig. S9). Besides the different equilibrium forms, no phase II metabolizing products were identified by LC–MS analysis (Fig. 6).

The analysis of the cell medium (DMEM with 2% FBS) containing the different compounds after 4 h in contact with cells at 37 °C (fraction removed from each well) demonstrated that the species present were the flavylium cation and chalcone species (Fig. 6). This occurs due to the fact that these pigments present a multistate of equilibrium species at pH 7.4, in the case of the three promising amino-based flavylium compounds, the positively charged flavylium is partially converted into yellow/colorless neutral forms (Fig. S5). Considering the different colors presented by the different equilibrium forms and the importance of this feature to their suitability for PDT, it is important to clarify which of these forms is able to be absorbed/absorbed and hence responsible for the cytotoxic effect.

Conversely to what was observed in the medium, the only species that was adsorbed/absorbed in cells was the flavylium cation (blue form). This result is very important as it suggests that the species that is activated by white light is mainly the flavylium cation, which present a maximum absorption wavelength near the red region at 596 nm, 672 nm and 575 nm, corresponding to 7NEt_2_4′NMe_2_, 7NEt_2_st4′NMe_2_, 7NEt_2_4′NH_2_, respectively. This interaction could be favored by the positive charge of this form and the negative charge of the hydrophilic polar heads of phospholipids.

### Reactive oxygen species (ROS)

The measurement of ROS is crucial to understand the photoactivation experiments and the potential role of the different compounds for PDT, once both types of PDT mechanisms involve the production of these species. For the measurement of ROS, cells were treated with the compounds in the same conditions as for the photoactivation experiments (4 h of incubation followed by dark or white light irradiation) and after this, DCF-DA was added. In the presence of ROS, this molecule is reduced and emits fluorescence at a specific wavelength. The signal for the control cells (cells without any treatment but in the presence of DCF-DA) was considered as 100% of ROS production. This is important once cells normally produce a basal and controlled level of ROS, crucial for their growth and by considering this as 100% it is possible to understand a reduction/stimulation without any previous stimulation (as in the case of the positive control where cells were stimulated with H_2_O_2_ for 30 min prior to the addition of the fluorescence probe). It was also important to select the ideal concentration of each compound to evaluate the modulatory effect of compounds, and by this, it was crucial to ensure that the cytotoxicity of compounds can be neglected in all the tested conditions but also that it was same for all the compounds tested. In this perspective, the applied concentration for all compounds was defined at 25 μΜ during the 4 h incubation period.

**Figure 7:**
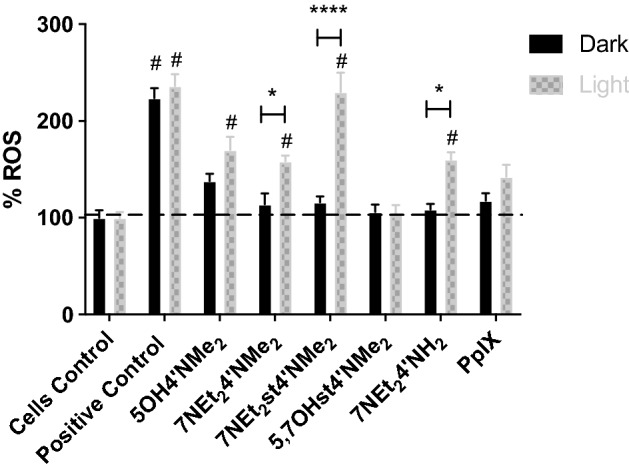
Production of reactive oxygen species by the different compounds at 25 μM upon white light irradiation (6.6 J cm^−2^) or in dark maintenance. As cells control, cells were not treated with any compound but the addition of DCF-DA was performed. As positive control the cells were treated with H_2_O_2_ (100 μM) 30 min prior to the addition of DCF-DA to induce a high production of ROS. Each value represents the mean ± SEM (n = 8–16). *Means treatment in dark significantly different to the treatment with the white light (*p value < 0.05; ****p value < 0.0001). ^#^Means significantly different from the respective control cells (cells without any concentration of compound): dark or white light treatment. To perform both statistical analysis, two-way ANOVA with Sidak’s multiple comparisons test was performed.

The first data that could be taken from Fig. 7 was the validation of the experiment, revealed by the results of the positive control for both dark and light irradiation treatments. Furthermore, the results suggest that light itself was not able to induce the production of ROS, as revealed by the lack of significant differences between dark and light irradiation treatments for the positive control.

This result is important to understand the role of the different compounds once it shows that light activates photosensitizers rather than directly activate cell biomolecules, which is in agreement with PDT principles. It could also be observed the lack of significant differences between control cells and cells incubated with compounds for the dark treatment, which suggests that the compounds did not induced the production of ROS in this condition. On the other hand, in the case of light irradiation, some compounds were able to significantly induce the production of these species as revealed from the significant differences found when comparing to control cells.

Although for 5OH4′NMe_2_ compound no significant differences were found between dark and light treatments, if one compares with the control cells it is possible to observe that in the case of light irradiation the production of ROS was significantly increased. To understand this, it is important to recall the photoactivation results for this compound. At an applied concentration of 25 μΜ, although this compound presented a significant toxicity in both treatments, it was possible to observe a significant reduction (*p < 0.05) of cell survival upon light irradiation treatment when compared to dark condition (Fig. 5). This may explain the observed result for ROS production, and also suggest that 5OH4′NMe_2_ seems to have a thin-line dose–response behavior for the modulation of ROS where it may induce cytotoxicity by other means rather than the stimulation of ROS metabolic pathways especially for higher concentrations.

Regarding 5,7OHst4′NMe_2_, this compound did not modulate the production of ROS either negatively or positively. This fact may explain the observed results for the photoactivation experiments.

With respect to 7NEt_2_4′NMe_2_, 7NEt_2_st4′NMe_2_ and 7NEt_2_4′NH_2_, data show a positive modulation of ROS production upon light irradiation comparing to cells control and dark conditions. This suggests that light irradiation induces activation of these compounds to initiate ROS metabolic pathways exacerbation. Although for PpIX no significant differences were observed in any conditions, the trend observed is similar to the other photoactivated compounds, which is in line with the described PDT role for this compound^27^. DCF-DA has different affinities for each reactive oxygen species, and although our results suggest the photoactivation of compounds towards PDT, additional experiments are necessary to assess the specific ROS metabolic pathways.

ROS production seems to depend on the same specific structural features as reported for the photoactivation experiments, which may be ranked as follows: C7-diethylamino > C4′-dimethylamino > C2-stryryl. To understand the mechanism of ROS production, we selected the most promising compounds: 7NEt_2_4′NMe_2_, 7NEt_2_st4′NMe_2_ and 7NEt_2_4′NH_2_.

### Photoactivation mechanism

**Figure 8:**
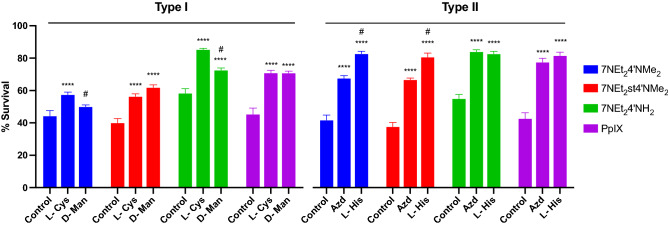
Mechanism of photoactivation of the compounds determined by the presence of different quenchers for type I and type II. As control the compounds were irradiated with light (6.6 J cm^−2^). For the treatments, the experiments followed the same conditions as for the photoactivation procedures but in the presence of the different quenchers (10 mM), individually. All the compounds were tested at a concentration of 50 μΜ. Each value represents the mean ± SEM (n = 8–24). *Means significantly different to the treatment control with the white light (****p value < 0.0001). ^#^Means significantly different from the respective equivalent quencher for the same compound. To perform both statistical analysis, two-way ANOVA with Sidak’s multiple comparisons test was performed. *L-Cys* l-cysteine 10 mM; *D-Man* d-mannitol 10 mM; *Azd* sodium azide 10 mM; *L-His* l-histidine 10 mM.

The photoactivation mechanisms of the most promising compounds were determined using different quenchers: sodium azide and l-histidine for singlet oxygen and l-cysteine and d-mannitol for other reactive oxygen species. Type I involves the transfer of electrons to the surrounding cellular biomolecules leading to the formation of free radicals, which is then followed by the production of ROS such as superoxide radicals (O_2_^−**·**^) and hydroxyl radicals (HO·). Instead, type II involves the activation of the ground state oxygen (^3^O_2_) to produce singlet oxygen (^1^O_2_). Figure 8 shows data obtained for the optimized experimental conditions (compounds at 50 μΜ and quencher at 10 mM in co-incubation during 4 h, followed by removal of compounds and addition of quencher at 10 mM to cells prior dark/light irradiation treatments). A significant reduction of cellular damaging in the presence of all the quenchers was observed for all compounds, which indicates that they respond either to type I, type II or both types of PDT photoactivation. Besides, type II quenchers seem to revert the cytotoxic action of the compounds with a higher efficacy when compared to type I for 7NEt_2_4′NMe_2_, 7NEt_2_st4′NMe_2_ and PpIX. The exception was 7NEt_2_4′NH_2_ where small differences in the survival rates were found for both mechanisms.

7NEt_2_4′NMe_2_ showed an increase of survival of 6 and 13% for d-Mannitol and l-Cysteine, respectively, and of 27 and 44% for sodium azide and l-histidine, respectively. Regarding 7NEt_2_st4′NMe_2_, the survival rate was 16 and 22% for l-cysteine and d-mannitol, respectively, and 31 and 46% for sodium azide and l-histidine, respectively. 7NEt_2_4′NH_2_ showed an increase of cells survival of 14 and 27% for d-mannitol and l-cysteine, respectively, and 31 and 33% for sodium azide and l-histidine, respectively. With respect to PpIX, the improvement of survival was 26 and 27% for l-cysteine and d-mannitol, respectively, and 37, 42% for sodium azide and l-histidine, respectively.

These results agree with the photoactivation process with preferential production of oxygen singlet for the experimental conditions used. Type I phototoxicity was also evident, suggesting a strong potential of these compounds to react with different reactive oxygen species, which represents an advantage in cells with low concentrations of triplet oxygen. Moreover, these results also corroborate with the previously discussed ROS experiments.

It was also observed that PpIX, a well-known PDT photosensitizer, participates in both types of cellular damaging, although, as expected, with a higher susceptibility to type II quenchers. This shows that these molecules are effective and comparable to established photosensitizers for PDT.

## Conclusion

The search for new alternative PS to the ones usually used has gaining importance over the last few years. Bearing this, natural-inspired molecules are the source of investigation such as the bio-inspired flavylium compounds herein described.

The chemical synthesis of such compounds was elaborated to match specific conditions such as their absorption maxima and the presence of bifunctional groups described to act in PDT. In fact, the results showed that the presence of the diethylamino functions in the core structure is crucial for the role of the compounds. It was possible to observe significant Φ_f_ for 3 compounds (7NEt_2_4′NMe_2_, 7Net_2_st4′NMe_2_ and 7Net_2_4′NH_2_), which were also the ones that presented photoactivated bioactivities in the different experiments for PDT. The results demonstrated that there seems to exist an inverse correlation between photoactivation efficiency and quantum yield for these set of compounds, which may be explained by the fact that with higher Φ_f_ the phosphorescence is lower, and consequently the triplet state condition is reduced diminishing the direct action of the compounds by type II mechanism.

Another important observation is the fact that the presence of the hydroxyl group at C5 position induced non-photoactivated cellular toxicity. On the other hand, the compounds with the best photoactivation results all present a diethylamino moiety at C7 and no hydroxyl group. Furthermore, the presence of two absorption maxima around 450 nm and 600–650 nm is common for the three photoactivated compounds. This is crucial for the therapeutical applications of PDT, which ideally makes use of higher wavelengths that can deeper penetrate tissues.

The results showed that all the compounds with a higher photoactivation showed a stimulation of ROS production in HaCaT cells when compared to the control, in the selected concentration, where the cytotoxicity in the dark was reduced in all cases. Once again, 7NEt_2_4′NMe_2_, 7NEt_2_st4′NMe_2_ and 7NEt_2_4′NH_2_ showed the highest yield in ROS production upon light stimulation, which confirms the induction of oxidative stress by these compounds upon light activation. The PSs can act by two distinct pathways upon activation by light. In the presence of specific quenchers, and due to the short lifetimes of the species generated during photoactivation of the photosensitizers, it is expected that the cell viability becomes higher in comparison to the absence of such quenchers. In this case, it would be expected to observe similar cell survivals when comparing dark and light treatments. At the selected concentration of 50 μM, for all the tested compounds, is clear an improvement of cell survival upon photoactivation. The compounds behavior differed for the type of mechanism potentially involved. There was no clear relation between the structural differences and the mechanism of action, however, it is clear that these compounds and this structural basis function at both type I and II PDT mechanisms.

Overall, this work allowed to characterize 3 new compounds suitable to be used in photodynamic therapy opening new ways and alternatives for the existing options in the market.

## Supplementary Information

Supplementary Figures.
