# Supplementary material for: Photoactivated cell-killing amino-based flavylium compounds
Source: Sci Rep. 2021 Nov 9;11:22005. doi: 10.1038/s41598-021-01485-y (PMC8578629; doi:10.1038/s41598-021-01485-y)
Supplement: Supplementary file 1 — Supplementary Figures. [file 41598_2021_1485_MOESM1_ESM.docx]

**Supplementary Information**

**Photoactivated cell-killing amino-based flavylium compounds**

Hélder Oliveira, Paula Araújo, Ana Rita Pereira, Nuno Mateus, Victor de Freitas, Joana Oliveira^*^, Iva Fernandes^*^

REQUIMTE – Laboratório Associado para a Química Verde, Departamento de Química e Bioquímica, Faculdade de Ciências, Universidade do Porto, Rua do Campo Alegre, 687, 4169-007 Porto, Portugal, *jsoliveira@fc.up.pt; *iva.fernandes@fc.up.pt

Keywords: Photodynamic therapy; amino-based flavylium; amino-based styryl-flavylium; singlet oxygen; skin

Figure S1. HPLC chromatogram profile of compounds: 5-hydroxy-4'-(dimethylamino)-benzopyrylium (5OH4'NMe_2_); 7-diethylamino-4'-dimethylamino-benzopyrylium (7NEt_2_4'NMe_2_), 7-diethylamino-4'-amino-benzopyrylium (7NEt_2_4'NH_2_), 7-diethylamino-2-(4'-dimethylaminostyryl)-benzopyrylium (7NEt_2_st4'NMe_2_) and 5,7-dihydroxy-2-(4'-dimethylaminostyryl)-benzopyrylium (5,7OHst4'NMe_2_).

a) DMEM_2% FBS_w/o_PR

b) DMSO

Figure S2. Absorbance spectra of 5-hydroxy-4'-(dimethylamino)-benzopyrylium (5OH4'NMe_2_); 7-diethylamino-4'-dimethylamino-benzopyrylium (7NEt_2_4'NMe_2_), 7-diethylamino-4'-amino-benzopyrylium (7NEt_2_4'NH_2_), 7-diethylamino-2-(4'-dimethylaminostyryl)-benzopyrylium (7NEt_2_st4'NMe_2_) and 5,7-dihydroxy-2-(4'-dimethylaminostyryl)-benzopyrylium (5,7OHst4'NMe_2_), in a) DMEM with 2% FBS without phenol red (DMEM_2% FBS_w/o_PR) and b) DMSO.

Figure S3. Fluorescence spectra of 5-hydroxy-4'-(dimethylamino)-benzopyrylium (5OH4'NMe_2_); 7-diethylamino-4'-dimethylamino-benzopyrylium (7NEt_2_4'NMe_2_), 7-diethylamino-4'-amino-benzopyrylium (7NEt_2_4'NH_2_), 7-diethylamino-2-(4'-dimethylaminostyryl)-benzopyrylium (7NEt_2_st4'NMe_2_), 5,7-dihydroxy-2-(4'-dimethylaminostyryl)-benzopyrylium (5,7OHst4'NMe_2_) and PpIX. Each spectrum was obtained after normalizing the solution to an absorption bellow 0.1 and by exciting the samples at the maximum absorption wavelength determined for DMSO.

Figure S4. UV-Vis spectra of the selected light sources for the photoactivation experiments.

Figure S5. Chemical reaction network established by 2-phenyl-1-benzopyrilium cations in slightly acidic aqueous solution (5<pH<7). Acid-base equilibrium only observed in case of R_3_=R_4_=OH. R_1_=R_2_= CH_3_, H. In the case of the zwitterionic base R_3_=R_4_= O^-^

Figure S6. HaCat cells survival in the presence of the different compounds upon irradiation with white light (6.6 J cm^-2^) or maintained in the dark. As a control for the experiment, protoporphyrin IX (PpIX) was used. The results are presented as the logarithmic function of the different tested concentrations (0.00-100.00 μM). Each value represents the mean ± SEM (n = 16-32). * means treatment in dark significantly different to the corresponding concentration of the treatment with the white light (*p value < 0.05; **p value < 0.01; ***p value = 0.001; ****p value <0.0001). # means treatment in dark concentrations significantly different from the control (cells without any concentration of compound). This last analysis was only done for dark treatment. To perform both statistical analysis the data was rearranged in grouped plots and after, two-way ANOVA with Sidak’s multiple comparisons test was performed.

Figure S7. Amount of absorbed and surface adsorbed compounds determined using trypan blue assay. Each value represents the mean ± SEM (n = 4-6).

Figure S8. Determination of the cellular uptake kinetics of the different compounds by HaCat cells. The compounds were added to the plates at a concentration of 100 μM and incubated for different time periods (15, 30, 60, 120, 180 and 240 minutes). The fluorescence intensity of each well was then determined at the different wavelength pairs specific for each tested compound (ex/em) (FlexStation 3 Multi‐Mode Microplate Reader). The fluorescence values were then used to plot into the corresponding calibration curves (see Materials and Methods section) and the uptake amounts were determined. Each value represents the mean ± SEM (n = 4-6). The results were plotted to a Michaelis-Menten curve.

Figure S9. Percentage of each species (flavylium cation, chalcone and dimer) in each of the compounds (7NEt_2_4'NMe_2_, 7NEt_2_st4'NMe_2_ and 7NEt_2_4'NH_2_) incubated in DMEM and DMEM-2 % FBS, at initial time (T0), 3h and 6h.
